# The Most Recent European and American Guidelines on Cardiac Rehabilitation: A Lack of Focus on Diaphragm Training

**DOI:** 10.7759/cureus.117884

**Published:** 2026-10-13

**Authors:** Bruno Bordoni, Bruno Morabito, Allan R Escher

**Affiliations:** 1 Physical Medicine and Rehabilitation, Foundation Don Carlo Gnocchi, Milan, ITA; 2 Physical Medicine and Rehabilitation, Associazione Italiana di Riabilitazione Cardiovascolare (AIRC), Milan, ITA; 3 Physical Medicine and Rehabilitation, School of Osteopathic Centre for Research and Studies, Milan, ITA; 4 Oncologic Sciences, University of South Florida Morsani College of Medicine, Tampa, USA; 5 Anesthesiology/Pain Medicine, H. Lee Moffitt Cancer Center and Research Institute, Tampa, USA

**Keywords:** airc, cardiac rehabilitation, cardiovascular diseases, global burden of disease, inspiratory muscle training

## Abstract

Cardiovascular diseases (CVDs) are the leading causes of death and morbidity worldwide. Patients with vascular and cardiac conditions undergo cardiac rehabilitation (CR), a process that may begin before surgery (less frequently), after invasive procedures, or as part of chronic disease management, with durations varying by healthcare facilities or national policies. CR is a valuable non-pharmacological intervention that supports patient health and the resumption of daily activities. These rehabilitation programmes are strongly recommended for cardiac patients and are structured according to guidelines from organisations such as the European Society of Cardiology (ESC) and the American Heart Association/American Association of Cardiovascular and Pulmonary Rehabilitation (AHA/AACPR). This editorial reviews the latest ESC and AHA/AACPR guidelines, emphasising physical activities such as endurance and resistance training, while highlighting the lack of detail on inspiratory muscle training (IMT). The editorial reiterates the Italian Association of Cardiovascular Rehabilitation (AIRC) proposals regarding IMT and calls for further clinical trials.

## Editorial

Cardiovascular diseases (CVDs) are the leading causes of mortality and hospitalisation worldwide. The Global Burden of Disease (GBD) 2023 study examined 18 CVDs across 204 countries and territories from 1990 to 2023, identifying 28 risk factors [1]. In 2023, it was estimated that 626 million people experienced hospitalisation or death (19.2 million) (Figure 1) [1].

**Figure 1:**
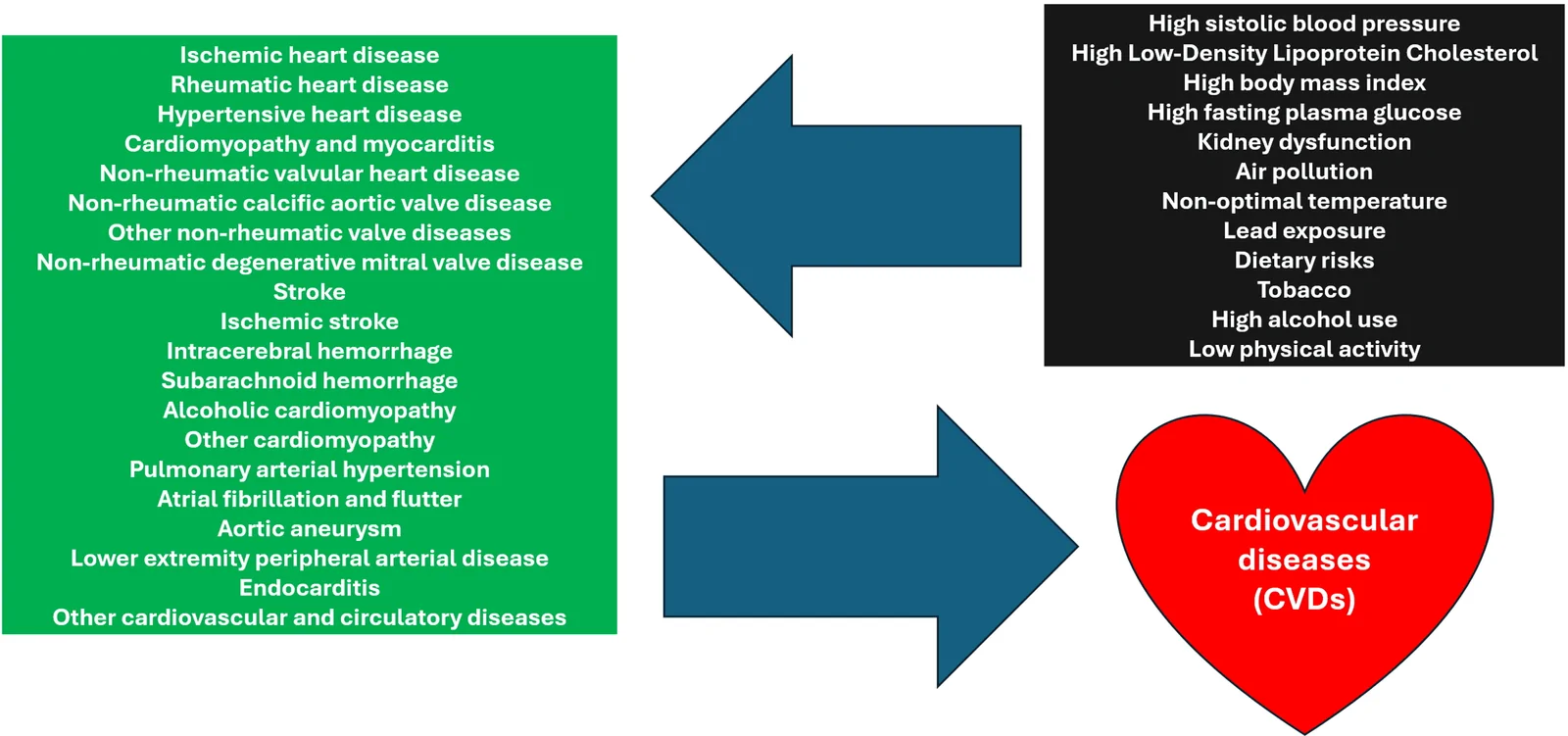
Cardiovascular diseases (CVDs) and the Global Burden of Disease (GBD) 2023 study The illustration summarises the cardiovascular diseases considered by the GBD (table on the left) and the major risk factors (table on the right). In 2023, it was estimated that 626 million people experienced hospitalisation or death (19.2 million). The image was created using Microsoft PowerPoint (Microsoft® Corp., Redmond, WA) by Bordoni Bruno. Reference: [1]

Cardiac rehabilitation (CR) is an interdisciplinary process for treating and preventing the recurrence of CVD, offering multiple benefits for patients, including reduced mortality and morbidity, improved quality of life, risk reduction, and faster recovery after an acute event or surgery [2,3]. Despite its proven efficacy, CR remains underutilised in both Europe and the United States [2,3]. CR is recommended as a pre-surgical strategy (pre-rehabilitation or phase 0), during the acute hospitalisation phase (inpatient rehabilitation or phase 1), in the post-acute phase (outpatient or supervised home-based care or phase 2), and for long-term independent maintenance (phase 3) [2].

In Europe, barriers - particularly those affecting women and older adults - prevent patients from accessing this treatment and prevention strategy [2]. In the United States, only one-third of patients actively participate in a CR programme, with particularly evident barriers to access for women, individuals with lower levels of education, and people of colour [4].

CR originated in the 1950s for patients who had suffered a heart attack - a time when absolute bed rest was prescribed, with only a few hours allowed in a chair. By the 1970s, recommendations had shifted to favour physical activity, albeit strictly within a controlled hospital setting, leading to the complexity of current guidelines [2-4].

Physical activity is the core of CR and provides benefits that cannot be achieved through pharmacological treatment alone [2,3]. Physical activity - such as endurance and resistance training (RT) - is safe whether performed in a controlled setting (phases 0 and 2) or remotely (via telemedicine) [2,3].

This editorial reviews physical activity recommendations from the guidelines of the European Society of Cardiology (ESC) and the American Heart Association/American Association of Cardiovascular and Pulmonary Rehabilitation (AHA/AACPR) - endorsed by the American College of Cardiology - while highlighting shortcomings in the organisation of physical activity programmes. The article notes the lack of emphasis on inspiratory muscle training (IMT) and reiterates the parameters established by the Italian Association of Cardiovascular Rehabilitation (AIRC) for more targeted diaphragm stimulation.

ESC guidelines

One of the novel features of these guidelines is the involvement of patients in their development using Patient-Reported Outcome Measures (PROMs) and Patient-Reported Experience Measures (PREMs). PROMs are standardised, validated questionnaires completed directly by patients to describe the impact of a disease and its treatment on their quality of life, symptoms, and daily functioning. PREMs are health indicators that measure the patient's perception of the quality of the experience throughout the care pathway [2]. These guidelines are not binding mandates but a framework to guide tailoring the therapeutic approach, with the final choice resting with the clinician and the patient's preferences [2].

Another aspect to consider is communication between the healthcare professional and the patient. It is estimated that between 40% and 80% of the information conveyed by the clinician to the patient is not retained [2]. ESC guidelines emphasise reiterating this information on multiple occasions so that the individual can assimilate new information regarding their health [2].

According to the ESC, CR is defined as follows: “Rehabilitation is a multimodal, patient-centred, collaborative process that has been shown to improve health outcomes and prognosis in patients with a broad spectrum of chronic conditions, including CVD and cancer” [2].

The types of patients considered suitable for participation in a CR programme are acute/chronic coronary syndrome, heart failure, left ventricular assist device, heart transplantation, valvular heart disease, congenital heart disease, atrial fibrillation, cardiac implantable electronic device, cardiomyopathies, cancer and cardiotoxic therapies, and pulmonary arterial hypertension [2].

How does the ESC committee envision CR? Once the patient’s clinical status and functional capacity have been assessed, CR can be initiated - provided clinical stability is maintained - within 14 days for non-cardiac surgery patients or within 28 days for cardiac surgery patients [2].

ESC highlights any risks and contraindications associated with CR, as well as contraindications to undergoing CR and exercise testing (cardiopulmonary exercise testing and/or the six-minute walk test (6MWT)). Increased risk associated with physical activity includes the following: New York Heart Association IV (more severe level of decompensation); significant blood pressure changes during physical activity (systolic); weight gain of more than 1.8 kg over the preceding one to three days; potentially dangerous ventricular arrhythmias occurring at rest or during exertion; resting heart rate or heart rate during physical activity exceeding 100 beats per minute; pre-existing physical limitations or medical conditions preventing physical activity; and unstable and/or untreated coronary stenoses.

Contraindications to physical activity and performance tests include potentially dangerous cardiac arrhythmias awaiting treatment, early physical activity following an acute event (less than two days), hemodynamic instability, ineffectively controlled systolic and diastolic blood pressure, atrioventricular block (three atrial beats for every conducted ventricular beat, 3:1, 4:1, or higher ratio), acute cardiac inflammation/infection, symptomatic aortic stenosis, intracardiac thrombus formation, hypertrophic cardiomyopathy with obstruction, and acute systemic disease.

Contraindications to physical activity include exercise intolerance, dyspnoea at rest, clinical instability, signs of ischemia on electrocardiogram/telemetry during physical exertion (<2 metabolic equivalents of task; <50 watts), uncontrolled diabetes, recent embolic event, acute thrombophlebitis, and newly diagnosed atrial fibrillation or atrial flutter.

For very frail patients, the ESC recommends initiating CR with five to 10 minutes of light active mobilisation, with the aim of gradually increasing physical activity. CR programmes should always be structured around modifications to specific training components - frequency, intensity, time, volume, and progression (FITT-VP) - to ensure variety and progression [2]. For moderate-intensity aerobic or endurance training (ET) (e.g., stationary bike, treadmill, walking), the ESC recommends 150-300 minutes per week, or 75-150 minutes for vigorous-intensity ET. For anaerobic activity or RT of moderate to high intensity, and for circuit resistance training (CRT), the ESC suggests an average of two days per week, with the optional inclusion of exercises to improve balance and flexibility (though these are not further specified) [2].

To summarise the parameters for ET, the recommended frequency is three to five days per week (two-thirds of total weekly physical activity), with each session lasting for 30-60 minutes. The recommended intensity is moderate or high (based on values derived from cardiopulmonary testing - considering heart rate or wattage - or using the Borg Rating of Perceived Exertion scale 6-20). ET may involve continuous activity or interval training, including stationary bike, treadmill, Nordic walking, and dancing. The recommended weekly calorie expenditure during active physical activity is 1000-2000 kcal.

The parameters for RT in CR according to the ESC include a frequency of two to three days per week (one-third of total weekly physical activity), with more than two sets for major muscle groups and 12-15 repetitions per exercise. The recommended intensity for upper limbs is 30-70% of the one-repetition maximum (1RM), while the recommended intensity for lower limbs is 40-80% of the 1RM.

The specific intensity level for ET (whether based on heart rate, oxygen consumption, wattage, or the Borg scale) is not defined. Instead, the guidelines specify the weekly caloric expenditure required for the exercise. This latter metric is not particularly practical to apply to patients or across all healthcare settings. For patients with hyperglycaemia or type 1 diabetes, the recommendation is to adjust Metabolic Equivalent of Task (MET) levels to structure training appropriately. However, calculating METs for each patient is neither simple nor straightforward.

There are no specifications for scheduling and adjusting the two main activities (ET and RT) throughout the week, nor is there a description of the number of sets for RT or the required rest intervals between sets. Furthermore, the guidelines do not indicate the duration over which training volume should be increased, nor do they specify whether a "deload" period should be included to prevent excessive fatigue or overtraining syndrome.

Regarding IMT, ESC guidelines are limited. The importance of combining IMT with an exercise training (ET/RT) regimen to improve well-being and performance parameters is acknowledged. In patients with heart failure, IMT can improve peak oxygen consumption (VO2peak) even without ET/RT [2]. There are no guidelines on IMT parameters for acute and chronic coronary syndromes. For chronic heart failure, the recommendation is to perform IMT three to four times a week, potentially progressing to daily breathing exercises. Sessions should last 20-30 minutes, with inspiratory resistance set at 30-60% of the maximal inspiratory pressure determined via spirometry [2].

AHA/AACPR guidelines

As with the ESC guidelines, physical activity is the cornerstone of CR [3]. Before undergoing CR, patients must undergo a clinical assessment, including instrumental and performance-based tests. Emphasis is placed on evaluating the patient's social and work environments to tailor physical activity and a healthy lifestyle to their individual needs [3].

Patient profiles eligible for CR under American guidelines differ from those under European guidelines. The types of patients considered suitable for participation in a CR programme are the following: myocardial infarction (within 12 months of the event), coronary artery bypass surgery, angioplasty and/or coronary stenting, heart valve repair or replacement, heart-lung transplantation, stable angina, and stable chronic heart failure.

These guidelines exclude patients with artificial hearts, atrial fibrillation, cardiac implantable electronic devices, cancer undergoing cardiotoxic therapies, or pulmonary arterial hypertension [3]. Contraindications to physical activity, or situations requiring extreme caution, are specified.

Contraindications to physical activity during CR, according to the guidelines of the AHA/AACPR, include the onset of angina, clinical instability, high systolic (over 240 mmHg) and/or diastolic (over 110 mmHg) blood pressure, and an ST-segment shift of more than 1 mm.

Other contraindications include reversible myocardial ischemia, moderate or severe abnormalities in cardiac wall motion, complex ventricular arrhythmias, second- or third-degree atrioventricular block, atrial fibrillation, supraventricular tachycardia, complex ventricular ectopy, and signs/symptoms of exercise intolerance.

According to these guidelines, the recommended parameters for ET include a frequency of three to five days per week. Moderate effort corresponds to 40-59% of heart rate reserve or an intensity of 12-13 on the Borg scale (6-20), while vigorous effort corresponds to 60-89% of heart rate reserve or an intensity of 14-17 on the Borg scale (6-20). The recommended duration is 20-60 minutes, including warm-up and cool-down activities. Training may be continuous or interval-based. Training volume may be adjusted per session or over the course of the week based on the following principles: frequency, intensity, and time. Volume may be expressed in terms of calories burned or metabolic equivalents (METs). The suggested progression involves increasing one of the components (frequency, intensity, or time). Duration may be increased by one to five minutes per session until the target duration is reached, while intensity may be increased by 5-10% per session or per week until the target intensity is reached.

ET can be performed using stationary bikes, treadmills, elliptical trainers, rowing machines, stair climbers, and recumbent steppers.

We do not know the optimal intensity or duration of physical exertion (which depends on the individual's clinical history), nor do we know how to manage heart disease in the presence of comorbidities and polypharmacy. Furthermore, the optimal duration of CR and the specific patient profiles that might derive the greatest benefit from it remain unknown [3].

Another new feature compared with the ESC guidelines is the highlighting of goals that cardiac patients should achieve through ET: increase in peak oxygen consumption of 15% or more compared to baseline values; increase in the capacity to sustain a workload equal to or greater than 40% of METs compared to values from the third rehabilitation session or the most recent session performed; increase of 10% or more in the distance covered during the 6MWT compared to the initial test.

Regarding RT parameters - as with the ESC guidelines - it remains unclear which exercises, movements, or training equipment are optimal, or whether RT is suitable for all patient categories (surgical vs. non-surgical, acute vs. chronic phases). For instance, how should RT exercises be implemented in a patient who has recently undergone a median sternotomy, and at what point post-surgery? These details remain undefined. The guidelines do not specify rest intervals between sets. However, they briefly recommend separating aerobic and anaerobic activities into distinct sessions [3].

To determine the appropriate lifting load and structure the training regimen (sets and repetitions), it is recommended that the 1RM be established once the patient is familiar with the exercise [3].

According to the guidelines of the AHA/AACPR, the recommended parameters for an RT regimen include a frequency of two to three non-consecutive days per week. The recommended intensity involves 10-15 repetitions per exercise at 40-60% of the 1RM, with a perceived exertion of 11-13 on the Borg scale (6-20). The recommended volume includes one to three sets per exercise, with eight 10 different exercises targeting major muscle groups.

There are several objectives associated with incorporating RT into CR: prevent and recover from a state of frailty; reduce the risk of accidental falls; successfully perform the Timed Up and Go (TUG) Test (completing the required sequence of movements in under 12 seconds); achieve an age-appropriate number of repetitions on the 30-Second Chair Stand Test, based on validated scoring criteria; perform the Four-Stage Balance Test with adequate balance; and achieve a score indicating functional mobility on the Berg Balance Scale (41-56).

A patient who has undergone CR, in which the programme duration is not precisely specified, should be able to perform approximately 150 minutes of moderate-intensity physical activity, or about 75 minutes of vigorous physical activity, per week [3].

American guidelines do not include IMT in CR.

IMT according to the Italian AIRC

Guidelines, which reflect the landscape of the scientific literature, lack studies on IMT and CR. The diaphragm performs extraordinary functions that extend far beyond simply bringing air into the lungs. Diaphragmatic dysfunction is frequently encountered as a result of iatrogenic damage from cardiac surgery, idiopathic causes, or pre-existing conditions. Notably, recovery of the diaphragm's neuro-mechanical function following cardiac surgery takes approximately four months [5]. Causes linked to the surgical process are manifold, ranging from the use of retractors, oxidative stress, and intraoperative mechanical ventilation to pre-surgical issues (such as pre-existing or idiopathic pathologies). Diaphragmatic dysfunction can cause symptoms, such as muscle weakness and dyspnoea, thereby limiting the patient's quality of life, performance capacity, and independence, while also increasing the duration of hospitalisation [5]. IMT improves diaphragmatic electromechanical function, respiratory function, and overall performance [5]. Relying solely on ET or RT is insufficient for comprehensive diaphragmatic stimulation [5].

To the best of the authors' knowledge, the literature reports no adverse effects associated with these respiratory exercises.

When planning a muscle training regimen, it is necessary to identify the most appropriate stimulation to ensure more comprehensive phenotypic engagement of muscle fibres. Eupnoeic breathing recruits red fibres (also known as aerobic, slow-twitch, or type I fibres), whereas sudden, strenuous actions involving the diaphragm recruit white fibres (also known as anaerobic, fast-twitch, or type II fibres) [5]. IMT must incorporate stimulation for both red and white fibres.

Inspiratory resistance is applied using mouthpiece-based devices (which vary by brand), with resistance modulated according to the subject's maximum inspiratory mouth pressure (PImax). The latter is determined via spirometry and can be considered conceptually equivalent to a 1RM [5]. In young subjects, inspiratory training at approximately 50% of PImax induces diaphragmatic fatigue, requiring a recovery period of about 30 minutes of eupnoeic breathing [5].

For skeletal muscle, and specifically to target slow-twitch (red) fibres, the literature recommends the following parameters: resistance should be set at <60% of maximum capacity (1RM), combining 15-20 repetitions or full movements (10-15 repetitions for weaker individuals), with 90-second rest intervals between sets and a maximum of two to three sets. The recommended training frequency is two to four days per week, or two to three days for weaker subjects [5]. For the stimulation of fast-twitch (white) fibres, different parameters are recommended: a load of 70-80% (relative to subjective 1RM), eight to 12 repetitions, a rest period of approximately three minutes between exercises, and two to three sessions per week [5].

In a previous article published in Cureus, AIRC proposed an organisational hypothesis for IMT modulation that can be applied during CR, regardless of the reason for starting rehabilitation [5]. These parameters stimulate red and white muscle fibres alternately, thereby avoiding fatigue and hyperinflation. The workload - specifically the inspiratory resistance settings - should be re-evaluated every one to two weeks. Furthermore, a week with significantly lower inspiratory resistance than the standard training regimen should be included to allow for physiological recovery and prevent overtraining syndrome [5]. The IMT protocol is divided into two daily sessions to avoid overlapping aerobic and anaerobic stimuli (split training) (Table 1) [5].

**Table 1:** Parameters for diaphragmatic stimulation via inspiratory muscle training (IMT), as previously published in an article in Cureus [5].

Inspiratory muscle split training for red fibres - afternoon	Inspiratory muscle split training for white fibres - morning
30% of maximal inspiratory pressures	50% of maximal inspiratory pressures
2-3 sets of 15 inhalations	2-3 sets of 8-12 inhalations
90-second rest between sets	3-minute rest between sets
2-3 days during the week	2-3 days during the week

How should IMT be incorporated into a CR programme that also includes RT and/or ET? As AIRC, we recommend performing IMT targeting slow-twitch (red) fibres before aerobic training and IMT targeting fast-twitch (white) fibres before anaerobic training. Ideally, keep ET and RT sessions separate, as suggested in American guidelines [3]. It should be clear that the proposed protocol is still hypothetical and requires further clinical validation before it can be considered a standard approach. These hypotheses require validation through further, larger-scale clinical trials.

As AIRC, we believe that IMT deserves greater consideration in the organisation of CR, and that ESC and AHA/AACPR guidelines should place greater emphasis on the diaphragm muscle in patients with heart disease, with more experimental studies conducted in this context. These indications are hypotheses derived from current literature and await further experimental investigation.
